# Consequences of polyploidy and divergence as revealed by cytogenetic mapping of tandem repeats in African clawed frogs (*Xenopus*, Pipidae)

**DOI:** 10.1007/s10344-023-01709-8

**Published:** 2023-07-21

**Authors:** Nicola R. Fornaini, Barbora Bergelová, Václav Gvoždík, Halina Černohorská, Vladimír Krylov, Svatava Kubíčková, Eric B. Fokam, Gabriel Badjedjea, Ben J. Evans, Martin Knytl

**Affiliations:** 1 Department of Cell Biology, Faculty of Science, Charles University, Viničná 7, Prague, 12843 Czech Republic; 2 Institute of Vertebrate Biology of the Czech Academy of Sciences, Brno, Czech Republic; 3 Department of Zoology, National Museum of the Czech Republic, Prague, Czech Republic; 4 Department of Genetics and Reproduction, CEITEC - Veterinary Research Institute, Hudcova 296/70, Brno, 62100 Czech Republic; 5 Department of Animal Biology and Conservation, University of Buea, PO Box 63, Buea, 00237 Cameroon; 6 Department of Aquatic Ecology, Biodiversity Monitoring Center, University of Kisangani, Kisangani, Democratic Republic of the Congo; 7 Department of Biology, McMaster University, 1280 Main Street West, Hamilton, ON L8S4K1 Canada

**Keywords:** Amphibians, Anura, snRNA, Histone H3, Allopolyploidization, In situ hybridization

## Abstract

Repetitive elements have been identified in several amphibian genomes using whole genome sequencing, but few studies have used cytogenetic mapping to visualize these elements in this vertebrate group. Here, we used fluorescence in situ hybridization and genomic data to map the U1 and U2 small nuclear RNAs and histone H3 in six species of African clawed frog (genus *Xenopus*), including, from subgenus *Silurana*, the diploid *Xenopus tropicalis* and its close allotetraploid relative *X. calcaratus* and, from subgenus *Xenopus*, the allotetraploid species *X. pygmaeus*, *X. allofraseri*, *X. laevis*, and *X. muelleri*. Results allowed us to qualitatively evaluate the relative roles of polyploidization and divergence in the evolution of repetitive elements because our focal species include allotetraploid species derived from two independent polyploidization events — one that is relatively young that gave rise to *X. calcaratus* and another that is older that gave rise to the other (older) allotetraploids. Our results demonstrated conserved loci number and position of signals in the species from subgenus *Silurana*; allotetraploid *X. calcaratus* has twice as many signals as diploid *X. tropicalis*. However, the content of repeats varied among the other allotetraploid species. We detected almost same number of signals in *X. muelleri* as in *X. calcaratus* and same number of signals in *X. pygmaeus*, *X. allofraseri*, *X. laevis* as in the diploid *X. tropicalis*. Overall, these results are consistent with the proposal that allopolyploidization duplicated these tandem repeats and that variation in their copy number was accumulated over time through reduction and expansion in a subset of the older allopolyploids.

## Introduction

Repetitive elements are genomic sequences that are present in multiple copies and are found in all eukaryotic genomes, but with varying abundances and genomic distributions. Content of repetitive DNA ranges from less than 10% in some fishes (e.g., *Tetraodon nigroviridis* and *Cynoglossus semilaevis*) and birds (e.g., *Phoenicopterus ruber*, *Struthio camelus*, *Haliaeetus albicilla*) to > 90% in some plants, such as *Allium cepa* (Chalopin et al. 2015; Chen et al. 2014; Zhang et al. 2014; Canapa et al. 2016; Fu et al. 2019).

Repetitive elements in eukaryotes are categorized into transposable elements (TEs) and tandem repeats. TEs, which are scattered throughout the genome, include retroelements/retrotransposons and DNA transposons. Retrotransposons are reversely transcribed to DNA and replicated using a copy and paste mechanism. Therefore, they may increase genome size (Yampolsky 2016; Clark et al. 2019). Among retrotransposons, the LINEs (Long Interspersed Nuclear Elements) are classified into many subcategories (Valente et al. 2011; Chalopin et al. 2015; Gama et al. 2022). In contrast, tandem repeats are found as tandem arrays or head-to-tail motifs from several to more than a hundred copies (Myers 2007). Tandem repeats are present in clusters within the genome on single chromosomal locus or multiple loci and include microsatellite, minisatellite, satellite DNA, ribosomal genes (minor 5S and major 45S), histone genes, and small nuclear RNA (snRNA) (Symonová et al. 2017b; Knytl et al. 2018a; Sember et al. 2020; Gazoni et al. 2021; Schott et al. 2022). These loci often undergo concerted evolution (Elder and Turner 1995; Liao 1999), causing intraspecific paralogous repetitive elements to be more similar to each other than to their orthologs, even when the origin of repetitive elements precedes speciation (Liao 1999). Repetitive loci on chromosomes show dynamic evolution in terms of where in the genome and how frequently they are found, and they often have a high rate of rearrangement (Bruschi et al. 2014; Liu et al. 2019).

Due to their repetitive nature, short read sequences typically pose challenges to mapping and repeat quantification, including using genomic data. However, localization of repetitive DNA can be achieved using fluorescent in situ hybridization (FISH) cytogenetic techniques. Cytogenetic mapping of repetitive sequences has been studied in teleost fishes (Bishani et al. 2021; Knytl et al. 2022), reptiles (Oliveira et al. 2021; Altmanová et al. 2022), birds (De Oliveira et al. 2017; Kretschmer et al. 2021), and mammals (Milioto et al. 2019; Gerbault-Seureau et al. 2018). In amphibians, cytogenetic mapping of repetitive sequences has identified inter- and intraspecific rearrangements and other features of karyotype evolution (da Silva et al. 2021; Phimphan et al. 2021; Guzmán et al. 2022).

**Fig. 1:**
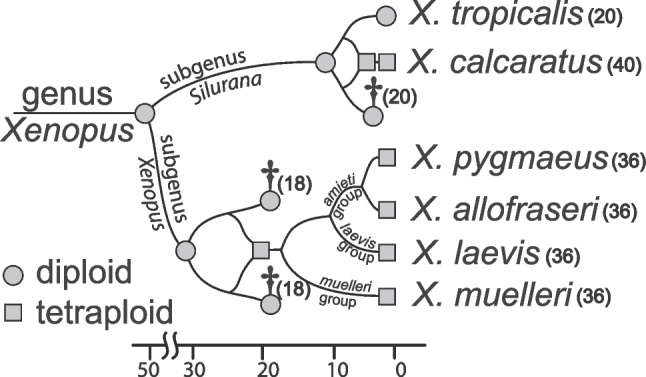
Inferred phylogenetic relationships and approximate divergence times among our focal taxa based on Evans et al. (2015), Session et al. (2016), and Feng et al. (2017). Allotetraploidization (depicted as reticulating lineages) occurred independently in each subgenus; inferred diploid lineages with no known extant diploid descendants are indicated with daggers. Time scale is in millions of years ago; chromosome numbers are shown in parentheses

African clawed frogs of the genus *Xenopus* (Pipidae) include almost 30 species and are divided into two subgenera (*Xenopus* and *Silurana*) (Evans et al. 2015) that each is sometimes considered to be a genus (e.g., Dubois et al. 2021). Subgenus *Silurana* includes four described species, the diploid *Xenopus* (*Silurana*) *tropicalis* and three allotetraploids *X. calcaratus*, *X. epitropicalis*, and *X. mellotropicalis*. For practical reasons, we use the name *Silurana* throughout this article, but present it at the subgenus level in accordance with Evans et al. (2015). Subgenus *Xenopus* includes 25 tetraploid, octoploid, or dodecaploid species (Tymowska 1991) that are divided into three species groups: *amieti*, *laevis*, and *muelleri* (Evans et al. 2015). In genus *Xenopus*, at least eight independent allopolyploidization events occurred, including at least one allotetraploidization event in each subgenus (Fig. 1; for an alternative scenario in *Silurana*, see Knytl et al. 2023). This variation in independently evolved ploidy levels offers a compelling opportunity to explore how tandem repeats evolve in the context of genome duplications.

About 34.5% and 40% of the *X. tropicalis* and *X. laevis* genomes are repetitive (Hellsten et al. 2010; Session et al. 2016). Ribosomal DNAs (rDNAs) have been cytogenetically mapped on *Xenopus* chromosomes (Schmid and Steinlein 2015; Knytl et al. 2017, 2023; Roco et al. 2021). In allotetraploid species of subgenus *Silurana*, rDNA FISH analysis identified nucleolar organizer region (NOR) on chromosome 7a where the letter “a” refers to the “a subgenome” within the allotetraploid genome (Knytl et al. 2017, 2023). That only one pair of homologous chromosomes contains the NOR locus in *Xenopus* allotetraploids suggests deletion of NOR on chromosome 7b (of the b subgenome) after allotetraploidization (Knytl et al. 2023) because a NOR would have been required in each diploid ancestral species. There is limited cytogenetic information from other repetitive sequences in *Xenopus*, and thus, it is also not known whether non-rDNA repeats vary among *Xenopus* species.

Small nuclear RNAs are components of the spliceosome that perform pre-mRNA splicing. The spliceosome is composed of five RNA tandem-repeat units (U1, U2, U4, U5, and U6 snRNAs) (Valadkhan 2005). Histones are components of the nucleosome that are present in tandem repeat families of five major genes: H1/H5, H2A, H2B, H3, and H4. Here, we used FISH for mapping U1 and U2 snRNA and histone H3 loci on chromosomes from six species of *Xenopus*, including members of both subgenera which diverged from one another approximately 45–50 Mya (Session et al. 2016; Feng et al. 2017). The allotetraploidization event in subgenus *Xenopus* is estimated to have occurred earlier (17–18 Mya) than the one in subgenus *Silurana* and the onset of the polyploid radiation in subgenus *Xenopus* at approximately 17 Mya (Session et al. 2016). Specifically, we examined the diploid *X. tropicalis* and its tetraploid close relative *X. calcaratus*, which are from subgenus *Silurana*, and the allotetraploids *X. pygmaeus*, *X. allofraseri* (both from the *amieti* species group), *X. laevis* (*laevis* species group), and *X. muelleri* (*muelleri* species group) which belong to subgenus *Xenopus* and arose from the older allotetraploidization. In the subgenus *Xenopus*, the L and S subgenomes diverged from one another about 30–35 Mya, whereas the subgenomes a and b in the tetraploid *Silurana* diverged from one another about 10 Mya (Evans et al. 2015; Session et al. 2016). With an overarching goal of exploring the effect of polyploidization and divergence on the evolution of tandem repeats, we used cytogenetic methods and a genome database to test the hypotheses that (i) the number of the repeat loci in diploid *X. tropicalis* is half that in the allotetraploid species, and (ii) the locations of repeat loci in the studied species are homologous.

## Materials and methods

### Primary cell cultures and metaphase spread preparations

Primary cell cultures were derived from laboratory strains of *X. tropicalis* (strain ‘Ivory Coast’), *X. laevis*, and *X. muelleri*, and wild-derived stains of *X. calcaratus* and *X. allofraseri* from Cameroon (Bakingili, where they occur in syntopy, 4.0684°N, 9.0682°E) and *X. pygmaeus* from the Democratic Republic of the Congo (Kokolopori, Yalokole, near Luo River, 0.2056°N, 22.8884°E). *Xenopus muelleri* animals were originally obtained from the Institute of Zoology at the University of Geneva (Switzerland). All species were bred at Charles University, Faculty of Science, Prague, Czech Republic. Briefly, tadpoles were anesthetized, hind limbs removed and homogenized (Sinzelle et al. 2012) in a cultivation medium (Knytl et al. 2017) modified according to Knytl et al. (2023). The explants were then cultivated at 29.5 °C with 5.5% CO_2_ for five days without disturbance. The media was then changed every day for one week. Passages were performed with trypsin-ethylenediaminetetraacetic acid (Knytl et al. 2017).

Chromosomal suspensions were prepared following Krylov et al. (2010) and stored in fixative solution (methanol/acetic acid, 3:1, v/v) at − 20 °C. For cytogenetic analysis, a chromosome suspension was dropped onto a slide (Courtet et al. 2001). Chromosome preparations were aged at − 20 °C for at least one week. For each experiment, mitotic metaphase spreads were counterstained with ProLong™ Diamond Antifade Mountant with the fluorescent 4′,6-diamidino-2-phenylindole, DAPI stain (Invitrogen by Thermo Fisher Scientific, Waltham, MA, USA). From ten to 20 metaphase spreads were analyzed per probe. Microscopy and processing of metaphase images were conducted using a Leica Microsystem (Wetzlar, Germany) as detailed in Seroussi et al. (2019).

### Fluorescent in situ hybridization with repetitive DNA probes

In order to generate probes for FISH, genomic DNA from *X. tropicalis* was used as a template for amplification of the repetitive small nuclear DNA regions (snDNA) U1 and U2, and histone H3. DNA was extracted from tadpole tail tissue using the DNeasy Blood and Tissue Kit (Qiagen, Hilden, Germany) according to manufacturer’s instructions. Primers used for amplification are listed in Table 1. The annealing temperature was 54 °C and the elongation step 30 s for all PCR reactions; other conditions for PCR amplification with PPP Master Mix (Top-Bio, Prague, Czech Republic) followed the manufacturer’s recommendations. Labeling PCR was performed as described in Knytl and Fornaini (2021). Digoxigenin-11-dUTP (Jena Bioscience, Jena, Germany) was used for U2 and H3 labeling, and biotin-16-dUTP (Jena Bioscience) was used for U1 labeling. *Xenopus tropicalis* U1 and U2 snDNA and H3 probes were then hybridized to chromosome spreads of *X. tropicalis*, *X. calcaratus*, *X. pygmaeus*, *X. allofraseri*, *X. laevis*, and *X. muelleri*. The procedures for hybridization mixture preparation, denaturation, and the subsequent overnight hybridization were described previously for rDNA FISH (Knytl et al. 2023). Post-hybridization washing and blocking reactions were performed as described for painting FISH in Krylov et al. (2010). Probe visualization was performed following Knytl et al. (2017). Slides were then de-stained according to the following protocol: nail polish was removed using xylene (2 min) and benzoin (2 min). Slides with cover slip were incubated in 4 × SSC/0.1% Tween for 10 min with agitation, and then cover slips were manually removed. Slides without cover slip were then incubated in 4 × SSC/0.1% Tween for 30 min with agitation followed by dehydration with methanol series (70, 90, 100% for 3 min each) and then air dried. After slide incubation in fixative solution for 30 min, slides were rinsed with distilled water, air dried, and then aged for 90 min at 60 °C.

**Table 1:** Genes used for FISH analysis, their GenBank accession numbers, lengths, sequences of primers, and studies in which primers were designed

Gene Symbol	Gene Name	Accession No	Length (bp)	Primer Sequence	Citation
U1	Small nuclear RNA U1	OQ714817	119	U1F: 5′-GCAGTCGAGATTCCCACATT-3′	Silva et al. (2015)
				U1R: 5′-CTTACCTGGCAGGGGAGATA-3′	
U2	Small nuclear RNA U2	OQ714818	177	U2F: 5′-ATCGCTTCTCGGCCTTATG-3′	Bueno et al. (2013)
				U2R: 5′-TCCCGGCGGTACTGCAATA-3′	
H3	Histone H3	OQ714819	364	H3F: 5′-ATGGCTCGTACCAAGCAGAC(ACG)GC-3′	Colgan et al. (1998)
				H3R: 5′-ATATCCTT(AG)GGCAT(AG)AT(AG)GTGAC-3′	

### Painting FISH

We used whole chromosome painting probes generated by laser microdissection of *X. tropicalis* chromosomes from previous study by Knytl et al. (2023). Whole chromosome painting probes from *X. tropicalis* chromosomes 1 and 8 were newly labeled with digoxigenin-11-dUTP (Krylov et al. 2010) and biotin-16-dUTP (both Jena Bioscience) (Knytl et al. 2023), respectively. De-stained slides (after FISH with the U1 and U2 probes) of *X. tropicalis*, *X. calcaratus*, and *X. laevis* were used for cross-species painting FISH (Zoo-FISH) with a digoxigenin-labeled probe according to the protocols described in Krylov et al. (2010) and modified in Knytl et al. (2017). Subsequently, slides were de-stained again and used for Zoo-FISH with biotin-labeled probe. Detection of signal was carried as detailed for double-color painting in Knytl et al. (2023). The *X. tropicalis* FISH experiments were performed in the reverse order from the other species (i.e., painting FISH first followed by de-staining and snDNA FISH).

## Results

### Sanger sequencing and BLAST searching

Amplification of the U1 and U2 snDNA and H3 nuclear genes yielded 119 and 177 and 364 bp long amplicons, respectively. Based on BLASTn searches of the *X. tropicalis* “Nigeria” strain genome, the U1 amplicon had 98.3% identity (query cover 100%) with the sequence of U1 spliceosomal RNA, LOC116408489 (accession number XR_004220992.1); U2 amplicon had 98.3% identity (query cover 100%) with U2 spliceosomal RNA, LOC116407440 (accession number XR_004220346.1); and the H3 amplicon had 97.2% identity (query cover 98%) with histone H3, LOC100497127, mRNA (accession number XM_012953339.3). Our sequences were deposited to the NCBI GenBank database: U1 snDNA accession number OQ714817, U2 snDNA accession number OQ714818, H3 accession number OQ714819.

### FISH with U1 and U2 snDNA probes and their genomic locations in available Xenopus genome assemblies

**Fig. 2:**
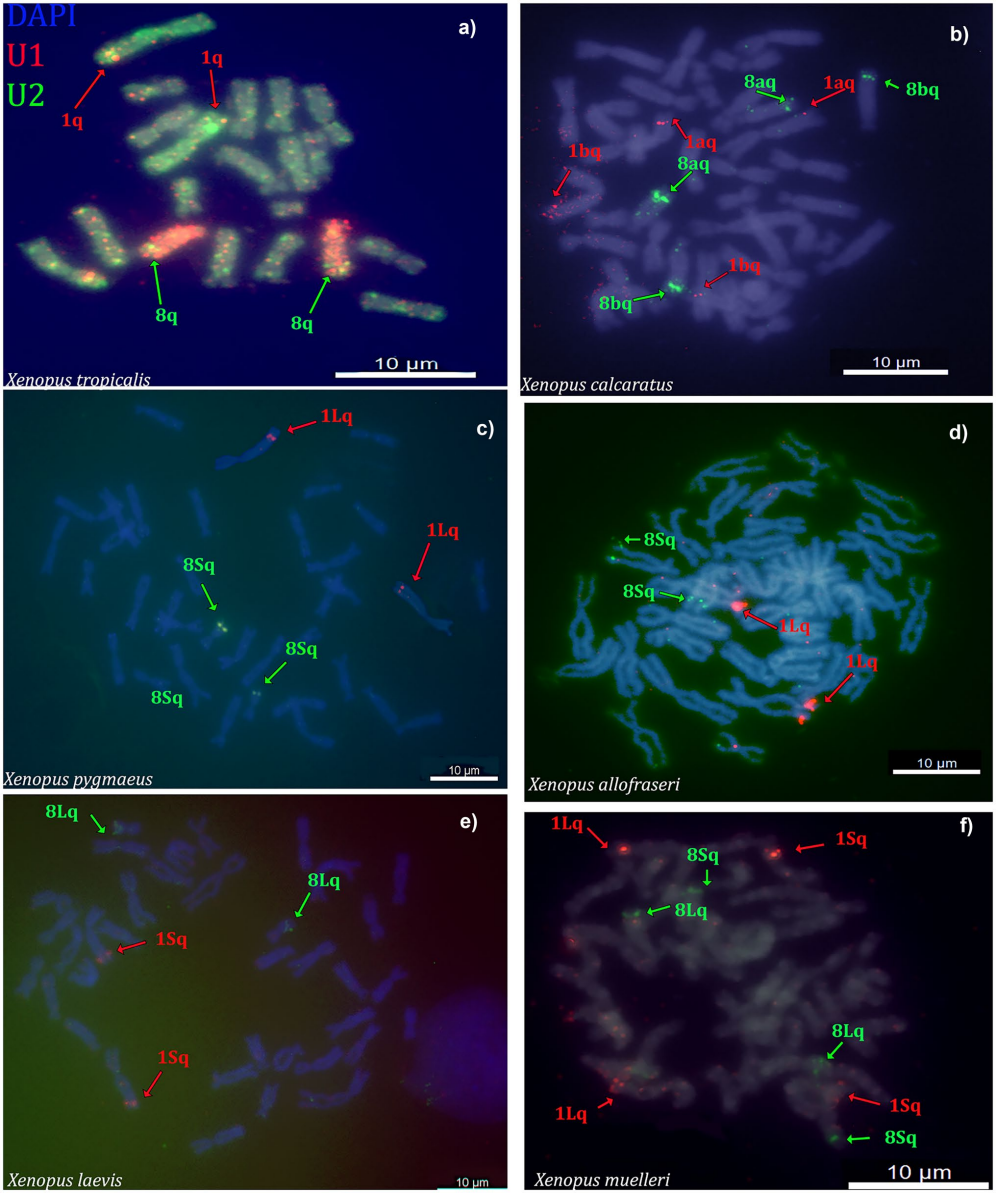
Double-color FISH with U1 and U2 snDNA probes. The U1 probe (red) reveals one clear signal (= a pair of homologous chromosomes) in **a** *X. tropicalis*, **c** *X. pygmaeus*, **d** *X. allofraseri*, and **e** *X. laevis*, while the same FISH shows two signals, each within homoeologous chromosomes in **b** *X. calcaratus* and **f** *X. muelleri*. The U2 (green) probe shows one signal in **a** *X. tropicalis*, **c** *X. pygmaeus*, **d** *X. allofraseri*, and **e** *X. laevis*, while the U2 probe shows two signals, each of them within homoeologous chromosomes in **b** *X. calcaratus* and **f** *X. muelleri*. Green and red arrows correspond to the U2 and U1 repeat loci, respectively. Painting probes were used for identification of chromosomes 1 (green) and 8 (red) in **a** *X. tropicalis.* Chromosomes were counterstained with DAPI in blue/gray. Scale bars represent 10 µM

When hybridized on the diploid species *X. tropicalis*, the U1 and U2 snDNA probes had intense signals on the distal region of the long (q) arm of chromosomes 1 and 8 (Fig. 2(a)), respectively. The number of copies was determined based on matches at least 50% of the query sequence length and 85% of the query sequence identity. BLAST results using the U1 sequence as a query to the *X. tropicalis* genome identified ~ 20 copies of U1 snDNA on chromosome 1. Using the U2 sequence as a query, we identified ~ 40 copies on *X. tropicalis* chromosome 8. In the allotetraploid species *X. calcaratus*, the U1 and U2 snDNA probes hybridized to the q arms of the chromosomes 1a, 1b and 8a, 8b (Fig. 2(b)), respectively. Chromosomes 1a and 1b are homoeologous to each other, and they are orthologous to *X. tropicalis* chromosome 1, and the mapped U1 region in *X. calcaratus* is thus homologous to the orthologous U1 region of *X. tropicalis* (both species have U1 gene on the distal part of the same chromosome). The U2 snRNA locus also was detected on both homoeologous chromosomes of *X. calcaratus* (chromosomes 8a and 8b) and in a homologous location to the orthologous U2 gene of *X. tropicalis.* For detailed definitions of homologous, homoeologous, and orthologous genes and chromosomes in *Xenopus*, see Song et al. (2021).

These U1 and U2 snDNA probes thus each hybridized to both homoeologous chromosomes in *X. calcaratus* as expected if copy number of each repeat was doubled by allopolyploidization. Similarly, in *X. muelleri*, U1 and U2 snDNA probes mapped to the expected homoeologous chromosomes (U1 on 1Lq and 1Sq; U2 on 8Lq and 8Sq; Fig. 2(f)).

In the allotetraploid species *X. pygmaeus*, *X. allofraseri*, and *X. laevis* only one signal was detected for U1 and U2 snDNAs. For the more closely related pair — *X. pygmaeus* and *X. allofraseri* — the U1 snDNA probe hybridized most conspicuously to the q arms of chromosome 1L and the U2 snDNA probe hybridized to the q arms of chromosome 8S (Fig. 2(c, d)). In *X. laevis*, the signal of the U1 and U2 snDNA probes was most conspicuous on the q arms of chromosomes 1S and 8L, respectively (Fig. 2(e)). Both U1 and U2 snRNA signals in *X. laevis* were located on chromosomes (1S and 8L) that are homoeologous to the chromosomes that bear U1 and U2 snRNA signals in *X. pygmaeus* and *X. allofraseri* (1L and 8S) and vice versa. The number of loci did not support the expectation that all tetraploid species should have twice as many snRNA loci as the diploid *X. tropicalis*, a result that could indicate copy number reduction or loss. Consistent with a scenario of reduction in copy number as opposed to complete loss, a BLAST search using U1 snDNA sequence as a query to the *X. laevis* genome recovered 11 copies on chromosome 1L and ~ 35 copies on chromosome 1S. Using the U2 sequence as a query against the *X. laevis* genome sequence, we identified ~ 40 copies on *X. laevis* chromosome 8L and 12 copies on chromosome 8S.

### Painting FISH

**Fig. 3:**
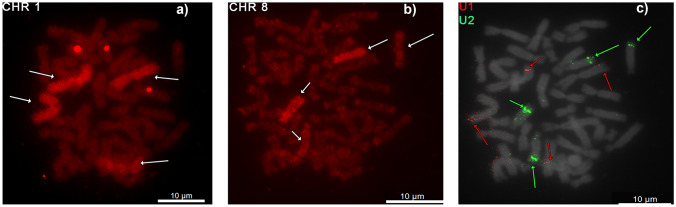
Sequential FISH in *X. calcaratus* chromosomes. Cross-species painting FISH experiments with the whole chromosome painting probes from *X. tropicalis* **a** chromosome 1 (XTR 1) and **b** XTR 8 highlight chromosome-bearing U1 and U2 snRNA loci, respectively. **c** snDNA FISH illustrates that the U1 locus (in red) is localized on *X. calcaratus* chromosomes 1 (XCA 1a and 1b) and the U2 locus (in green) on XCA 8a and 8b. **a** and **b** are shown in the red channel, **c** is merged in the red-green-blue (RGB) channels. **c** chromosomes were counterstained with DAPI in gray. Scale bars represent 10 µM

**Fig. 4:**
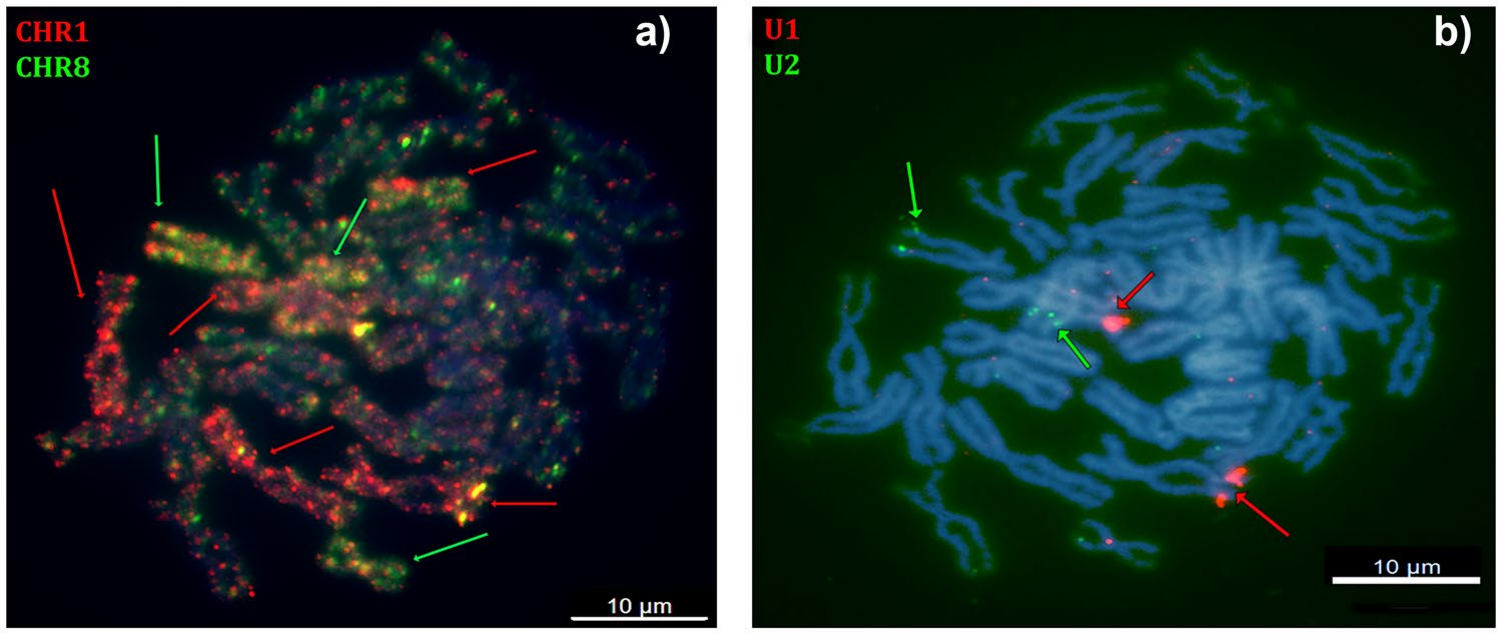
Sequential FISH in *X. laevis* chromosomes. Cross-species painting FISH experiments with whole chromosome painting probes from **a** XTR 1 (in red) and XTR 8 (in green) illustrate that **b** the U1 locus (in red) is localized on *X. laevis* chromosomes 1S (XLA 1S), and the U2 locus (in green) on XLA 8L. Both **a** and **b** are merged in the RGB channels. Chromosomes were counterstained with DAPI in blue. Scale bars represent 10 µM

To identify chromosomes bearing U1 and U2 loci, we employed FISH with the whole chromosome painting probes from *X. tropicalis* chromosomes 1 and 8. We successfully identified chromosomes 1 and 8 in *X. tropicalis* (Fig. 2(a)); chromosomes 1a, 1b, 8a, and 8b in *X. calcaratus* (Fig. 3); and chromosomes 1L, 1S, 8L, and 8S in *X. laevis* (Fig. 4). Homoeologous chromosomes of *X. calcaratus* and *X. laevis* were identified based on the intensity of the fluorescence signal (Knytl et al. 2023) and in the other studied species in subgenus *Xenopus* in which the painting FISH approach was not conducted; instead, homoeologous chromosomes were distinguished based on chromosome length (the L homoeologous chromosomes are longer than the S chromosomes; Matsuda et al. 2015; Session et al. 2016).

### FISH with histone H3 probe and its genomic locations in available *Xenopus* genome assemblies

**Fig. 5:**
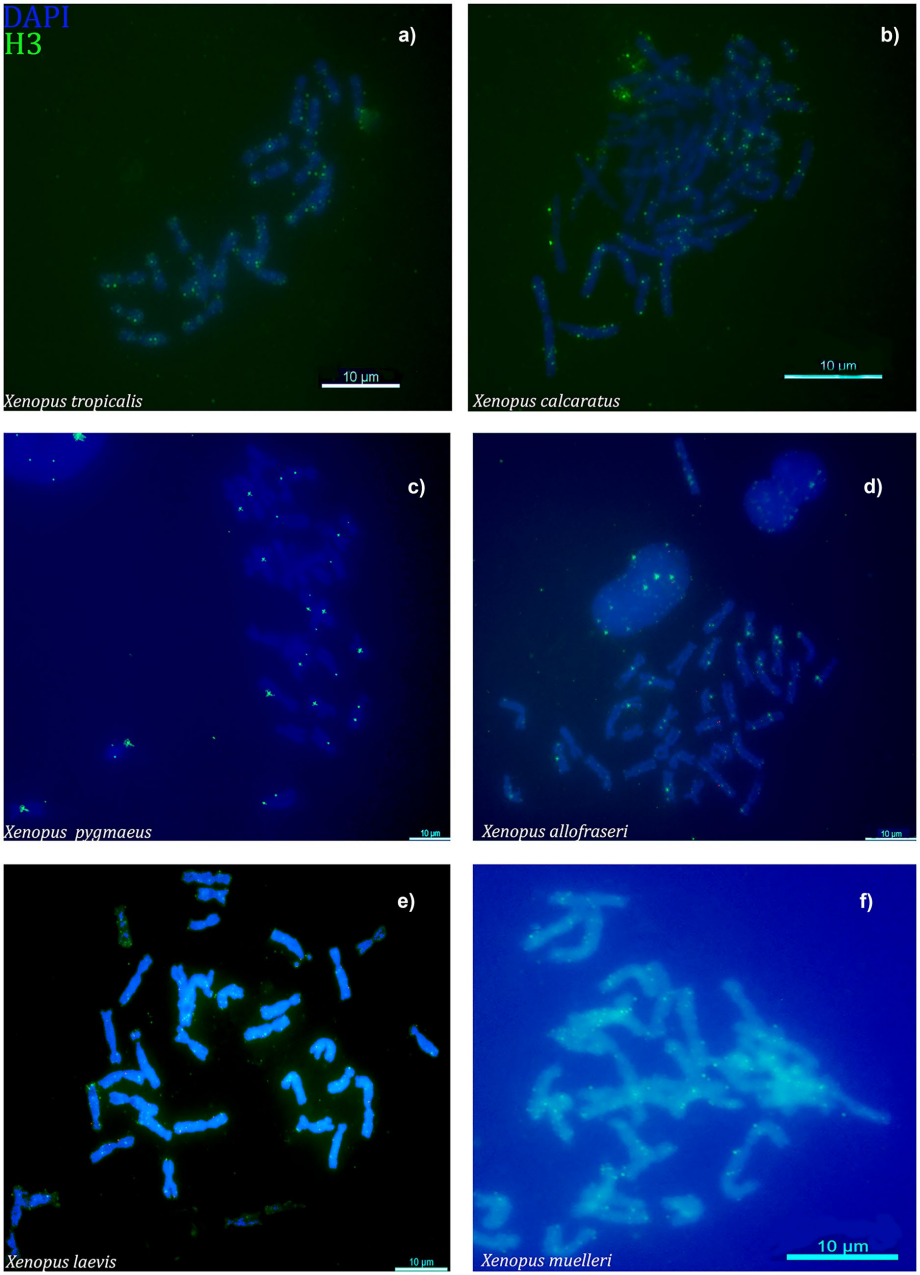
FISH with histone H3 probe. The probe (green) shows multiple signals in **a** *X. tropicalis*, **b** *X. calcaratus*, **c** *X. pygmaeus*, **d** *X. allofraseri*, **e** *X. laevis*, and **f** *X. muelleri*. Chromosomes were counterstained with DAPI in blue/gray. Scale bars represent 10 µM

We then hybridized H3 probe to chromosome spreads of *X. tropicalis*, *X. calcaratus*, *X. pygmaeus*, *X. allofraseri*, *X. laevis*, and *X. muelleri*. In all species, signals were present in small patches on multiple chromosomes (Fig. 5). We found signals in all chromosomes in *X. tropicalis*, *X. calcaratus*, and *X. muelleri*. However, *X. pygmaeus*, *X. allofraseri*, and *X. laevis* had signals on about half of chromosomes. The H3 probes mapped to telomeric and pericentromeric regions.

Based on BLAST searches, the H3 sequence occurs on *X. tropicalis* chromosomes 3 (11 hits), 6 (11 hits), and 9 (27 hits), and on chromosomes 5, 8, and 10, the matches were less than 50% of the query length. In *Xenopus laevis*, the H3 sequence occurs on chromosomes 3S (12 hits), 5L (6 hits), 5S (3 hits), 6L (2 hits), 6S (1 hit), 9_10L (15 hits), and 9_10S (5 hits). *Xenopus laevis *chromosomes 1L and 8L show some hits being less than 50% of the query sequence. All U1 and U1 snRNA and H3 loci mapped by FISH and BLAST are shown in Table 2.

**Table 2:** Numbers and chromosomal positions of U1 and U2 snRNA and H3 loci per haploid genome in diploid *X. tropicalis* (subgenus *Silurana*) and reduced genome (half of the somatic set) in allotetraploid *X. calcaratus* (subgenus *Silurana*), *X. pygmaeus*, *X. allofraseri* (both in the *amieti* species group), *X. laevis* (*laevis* species group), and *X. muelleri* (*muelleri* species group). The numbers of loci are given according to the FISH results and the genomic database. The XTR, XCA, XPY, XAL, XLA, and XMU abbreviations correspond to chromosome of *X. tropicalis*, *X. calcaratus*, *X. pygmaeus*, *X. allofraseri*, *X. laevis*, and *X. muelleri*, respectively. *NA* means that information is not available in the genome database

Species	Number of chromosomes	U1 snRNA by FISH	U1 snRNA genomic database	U2 snRNA revealed by FISH	U2 snRNA genomic database	H3 by FISH	H3 genomic database
*X. tropicalis*	*n* = 10	1 (XTR 1)	1 (XTR 1)	1 (XTR 8)	1 (XTR 8)	10	6
*X. calcaratus*	*n* = 20	2 (XCA 1a and 1b)	NA	2 (XCA 8a and 8b)	NA	20	NA
*X. pygmaeus*	*n* = 18	1 (XPY 1L)	NA	1 (XPY 8S)	NA	8	NA
*X. allofraseri*	*n* = 18	1 (XAL 1L)	NA	1 (XAL 8S)	NA	8	NA
*X. laevis*	*n* = 18	1 (XLA 1S)	2 (XLA 1L, XLA 1S)	1 (XLA 8L)	2 (XLA 8L, XLA 8S)	8	9
*X. muelleri*	*n* = 18	2 (XMU 1L and 1S)	NA	2 (XMU 8L and 8S)	NA	18	NA

## Discussion

Quantification and localization of repetitive sequences using high-quality genome sequencing and assembly is an expensive and challenging undertaking as compared to using multiple cytogenetic approaches for gene mapping (Knytl et al. 2018b; Symonová et al. 2017a). As an alternative, we cytogenetically mapped non-rDNA tandem repeats (U1 and U2 snRNA and H3 histone) to one diploid and five allotetraploid *Xenopus* species with the aim of testing how repetitive elements were affected by genome duplication and divergence. At least two allotetraploidization events occurred in genus *Xenopus* with respect to our focal species and both — including studied species and their diploid extinct or yet undescribed predecessors — are depicted in Fig. 1.

Barring loss or movement of tandem repeats, we expected allotetraploid species to have twice as many tandem repeats as the diploid *X. tropicalis*, and that the locations of these elements would be unchanged (i.e., on the same region of both homoeologous chromosomes). In general, the cytogenetic results recovered no evidence for long-range movement of the U1, U2 snRNA or H3 loci. In the allotetraploids *X. calcaratus* and *X. muelleri*, the observed number of signals for each of the U1 and U2 snRNA loci matched our expectation based on allopolyploidization. However, for the three other allotetraploids (*X. pygmaeus*, *X. allofraseri*, and *X. laevis*), this expectation was not supported because, although in homologous locations, we found the same number of these tandem repeats as in the diploid *X. tropicalis* (Table 2). Furthermore, the snRNA signals in *X. pygmaeus* and *X. allofraseri* were on a different subgenome (U1 on L, U2 on S) than in *X. laevis* (U1 on S, U2 on L). Overall, this is consistent with an increased variation in copy number over time following allopolyploidization. BLAST searches of the *X. laevis* genome recovered each of these loci in both subgenomes, and it appears that the lower-than-expected number of cytogenetic signals is thus a consequence of differences in copy number, at least in this species. That the signals in *X. pygmaeus* and *X. allofraseri* are on different subgenomes than in *X. laevis* suggests that changes in copy number have been an ongoing phenomenon during *Xenopus* diversification. During diversification of African clawed frogs, the ancestor of *X. muelleri* diverged from the common ancestor of *X. pygmaeus*, *X. allofraseri*, and *X. laevis* soon after allotetraploidization in subgenus *Xenopus*, around 17 Mya (Fig. 1; Evans et al. 2015; Session et al. 2016). Thus, a parsimonious interpretation of these results posits that changes in copy number arose independently in an ancestor of *X. laevis* and again in the most recent common ancestor of *X. pygmaeus* and *X. allofraseri*.

In amphibians, U2 snRNA tandem repeats have also been localized in the cycloramphid genus *Thoropa* (Cholak et al. 2020) and the leptodactylid genus *Leptodactylus* (Gazoni et al. 2021), and the H3 gene has been localized in the pipid genus *Pipa* (Zattera et al. 2020)*.* To our knowledge, there are no other cytogenetic studies that localize the U1, U2 snRNA, or H3 gene loci in this vertebrate group.

In frog genus *Thoropa*, U2 snRNA was detected cytogenetically on chromosomes 6 and 7 in some species, but others had U2 locus only on chromosome 6 or only on chromosome 7 (Cholak et al. 2020). The explanation for this variation might be that U2 loci are universally situated on both chromosomes 6 and 7 in all investigated *Thoropa* species but with different copy numbers on each of these chromosomes. We found similar variability in the location and number of FISH signals. Using BLAST, we identified locations where the sequence of our probe matched the *X. laevis* genome sequence and found that the number of U1 and U2 snRNAs detected by BLAST did not match the number of snRNAs detected by FISH. This inconsistency may be due to variation in the copy number of tandem repeats in each *X. laevis* subgenome and the lack of sensitivity of FISH to detect the locus with a low copy number of repeats. The U1 and U2 snRNA FISH signals were cytogenetically detected on those *X. laevis* chromosomes that contain ~ 15 and more copies of U1 and U2 repeats. In *Leptodactylus*, the U2 loci were identified on chromosome 6 in eight species, but one of them had signals on chromosomes 4, 6, 9, and 10 (Gazoni et al. 2021). These authors proposed that the additional signals on chromosomes 4, 9, and 10 can be the result of transposition by TEs followed amplification of the gene. We did not map TEs on *Xenopus* chromosomes and are thus unable to evaluate the possibility of TE transposition in this genus.

We also evaluated the effects of allotetraploidization and divergence on the evolution of H3 repeats. As expected, due to allotetraploidization, the allotetraploids *X. calcaratus* and *X. muelleri* possessed H3 repeats on twice as many chromosomes as the diploid *X. tropicalis*. However, in the allotetraploid *X. pygmaeus*, *X. allofraseri*, and *X. laevis*, we found signals on eight homologous pairs, which are fewer than expected based on allotetraploidization. This presumably is due to a decreased copy number or deletion of H3 repeats on several chromosomes, possibly in an ancestor of *X. pygmaeus*, *X. allofraseri*, and *X. laevis* based on phylogenetic relationships among these species. Despite the variability associated with allopolyploidization followed by divergence in evolution of tandem repeats, the relative dosages of signals of U1, U2, and H3 with respect to *X. tropicalis* are similar in each allotetraploid species we examined.

Until recently, there was only one study in which the H3 gene was mapped on amphibian chromosomes (Zattera et al. 2020). In *Pipa carvalhoi*, a representative of the same family as *Xenopus* but deeply phylogenetically divergent by about 115–120 My (Feng et al. 2017; Hime et al. 2021), H3 signals were detected on chromosomes 1, 5, 6, 8, and 9, and less intense signals were visible on other chromosomes. Multiple distributions of H3 locus on all chromosomes have been observed, for example, in insect species of the family Acrididae (Oliveira et al. 2011; Bueno et al. 2013) where the distribution of the H3 locus may be related to the transposition of 5S ribosomal RNA (rRNA). In *Xenopus*, 5S rRNA is situated on telomeres of multiple chromosomes (Knytl et al. 2017, 2023), and therefore, the observed distribution of the H3 locus on multiple *Xenopus* chromosomes may also be associated to the distribution of 5S rRNA.

### Co-localization of repetitive elements

An interesting finding that emerged from this study is a genomic association of various repetitive elements with rRNA; this can potentially provide evolutionary insights into genome organization. Synteny exists between As51 satellite DNA (originating from TEs) and NORs (Vicari et al. 2008) or between U1 snRNA and 5S rRNA in the characid fish genus *Astyanax* (Silva et al. 2015). One of possible roles of a co-localization can be a silencing of ribosomal genes by TEs (Vicari et al. 2008). All *Xenopus* species have NOR on a single homologous pair (Tymowska 1991). *Xenopus tropicalis* has NOR on chromosome 7, tetraploids from subgenus *Silurana* on 7a, and tetraploids from subgenus *Xenopus* on 3L (Tymowska 1991; Session et al. 2016; Roco et al. 2021; Knytl et al. 2023). We have not identified any snRNA loci on chromosomes 7, 7a, or 3L, and this finding argues against a genomic association between distinct NOR and snRNA repeats. The 5S ribosomal genes have been detected at the distal regions of the *X. mellotropicalis* and *X. calcaratus* (*Silurana* tetraploids) chromosomes 8bq (Knytl et al. 2017, 2023) which is the same position as the U2 snRNA locus in *X. calcaratus* (this study), supporting a genomic association between 5S rRNA and U2 snRNA. The NOR locus presumably was deleted on chromosome 7b in tetraploids of subgenus *Silurana* and on chromosome 3S in tetraploids of subgenus *Xenopus* (Session et al. 2016; Knytl et al. 2023). However, U1 and U2 loci map to both homoeologs in some tetraploids, and thus, deletion of snRNA in an ancestor of *Xenopus* tetraploids did not take place. There is the possibility that all our investigated *Xenopus* tetraploids have twice as many snRNA repeat loci as the diploid *X. tropicalis* but that these loci have unequal copy number within individual subgenomes, meaning that copy number of snRNA repeats was reduced in one subgenome (11 copies of U1 snRNA on *X. laevis* chromosome 1L is less than 20 copies on *X. tropicalis* chromosome 1; thus, the copy number of U1 snRNA on *X. laevis* chromosome 1L was reduced) but expanded in the other subgenome (35 copies of U1 snRNA on *X. laevis* chromosome 1S versus 20 copies in *X. tropicalis*)*.* Moreover, some tandem repeat loci maintain a stable number of copies, for example, 40 copies of U2 snRNA on *X. tropicalis* chromosome 8 and *X. laevis* chromosomes 8L). These stable copy numbers within the U2 gene are consistent with studies that observed a higher evolutionary conservation of the *X. laevis* subgenome L than subgenome S, compared to *X. tropicalis* genome (Session et al. 2016). Reduction, expansion, and stability in the copy number of non-rDNA tandem repeats and complete loss of rRNA locus are the possible events that occur after polyploidization (Session et al. 2016; Knytl et al. 2023). Genes retained as duplicates have distinct evolution leading to loss of function, subfunctionalization, or neofunctionalization and are under strong purifying selection (Force et al. 1999; Lynch et al. 2001), often followed by intra- and/or interchromosomal translocations, inversions, deletions, or degenerations (Evans 2007; Sémon and Wolfe 2007; Session et al. 2016; Knytl et al. 2017). Our study highlighted that copy number reduction and expansion of tandem repeats can be an important driver of evolution following allopolyploidization such as translocation, inversion, deletion, and degeneration.

## Conclusion

Repetitive elements are integral components of a genome, and their evolution can be affected by mechanism of polyploidization or divergence among species. We used cytogenetic approaches to study repetitive elements in six species of *Xenopus* frogs (one diploid, five allotetraploids) with an aim of localizing and quantifying tandem repeats in their genomes. For U1 and U2 snRNA, and H3 tandem repeats, the locations were generally homologous, but we detected variation in copy numbers that resulted from reduction and expansion after allotetraploidization. The dynamic evolution of tandem repeats was most apparent in allotetraploid species that arose from the older allotetraploidization event.

## Data Availability

The Sanger sequences that support the findings of this study are openly available in GenBank (accession numbers OQ714817-9).
